# Cell Wall Anchoring of the *Campylobacter* Antigens to *Lactococcus lactis*

**DOI:** 10.3389/fmicb.2016.00165

**Published:** 2016-02-18

**Authors:** Patrycja A. Kobierecka, Barbara Olech, Monika Książek, Katarzyna Derlatka, Iwona Adamska, Paweł M. Majewski, Elżbieta K. Jagusztyn-Krynicka, Agnieszka K. Wyszyńska

**Affiliations:** ^1^Department of Bacterial Genetics, Institute of Microbiology, Faculty of Biology, University of WarsawWarsaw, Poland; ^2^Department of Animal Physiology, Institute of Zoology, Faculty of Biology, University of WarsawWarsaw, Poland

**Keywords:** *Lactococcus lactis*, LPXTG cell wall anchor domain, *Campylobacter*, vaccine, chicken immunization

## Abstract

*Campylobacter jejuni* is the most frequent cause of human food-borne gastroenteritis and chicken meat is the main source of infection. Recent studies showed that broiler chicken immunization against *Campylobacter* should be the most efficient way to lower the number of human infections by this pathogen. Induction of the mucosal immune system after oral antigen administration should provide protective immunity to chickens. In this work we tested the usefulness of *Lactococcus lactis*, the most extensively studied lactic acid bacterium, as a delivery vector for *Campylobacter* antigens. First we constructed hybrid protein – CjaA antigen presenting CjaD peptide epitopes on its surface. We showed that specific rabbit anti-rCjaAD serum reacted strongly with both CjaA and CjaD produced by a wild type *C. jejuni* strain. Next, rCjaAD and CjaA were fused to the C-terminus of the *L. lactis* YndF containing the LPTXG motif. The genes expressing these proteins were transcribed under control of the *L. lactis* Usp45 promoter and their products contain the Usp45 signal sequences. This strategy ensures a cell surface location of both analyzed proteins, which was confirmed by immunofluorescence assay. In order to evaluate the impact of antigen location on vaccine prototype efficacy, a *L. lactis* strain producing cytoplasm-located rCjaAD was also generated. Animal experiments showed a decrease of *Campylobacter* cecal load in vaccinated birds as compared with the control group and showed that the *L. lactis* harboring the surface-exposed rCjaAD antigen afforded greater protection than the *L. lactis* producing cytoplasm-located rCjaAD. To the best of our knowledge, this is the first attempt to employ Lactic Acid Bacteria (LAB) strains as a mucosal delivery vehicle for chicken immunization. Although the observed reduction of chicken colonization by *Campylobacter* resulting from vaccination was rather moderate, the experiments showed that LAB strains can be considered as an alternative vector to deliver heterologous antigens to the bird immune system. Additionally, the analysis of the structure and immunogenicity of the generated rCjaAD hybrid protein showed that the CjaA antigen can be considered as a starting point to construct multiepitope anti-*Campylobacter* vaccines.

## Introduction

*Campylobacter* sp., members of Epsilonproteobacteria, are intestinal inhabitants of a various animal and avian species and, at the same time, are a major cause of human bacterial food-borne gastroenteritis; each year they are responsible for several 100 million cases of infection worldwide. The number of reported confirmed cases of human campylobacteriosis varies between countries and ranges between ten to more than 100 per 100,000 population (Kaakoush et al., 2015). In the EU in 2013, 214,779 cases were recorded (EFSA and ECDC, 2015). The number of *Campylobacter* genus species is growing constantly. Among at least 34 (http://www.bacterio.net/campylobacter.html\%20consulted\%20on\%2001/2016) species of the *Campylobacter* genus which have been described so far, the most prevalent species isolated from clinical cases of human campylobacteriosis are *C. jejuni* and *C. coli* (Robyn et al., 2015). Whereas in developing countries, the disease is endemic and affects mainly children, in industrialized countries most cases of the disease are mainly sporadic and are caused by the consumption of pathogen-contaminated, improperly prepared broiler meat. The gastrointestinal tract of infected broiler chickens contains a very high load of *C.* *jejuni* (Silva et al., 2011; Hermans et al., 2012; Powell et al., 2012). So, taking into consideration the broad consumption of poultry meat products, it has been established that the chicken reservoir is the main source of human campylobacteriosis. It was calculated that decreasing the count of *Campylobacter* in chicken intestines by 2 log_10_-units would lower the number of human campylobacteriosis cases 30-fold, and that a reduction by 3 log units should diminish the public health risk by at least 90% (EFSA Panel on Biological Hazards (BIOHAZ), 2011; Rosenquist et al., 2013).

Reduction of chicken colonization by *Campylobacter* can be achieved by vaccination, but an effective chicken vaccine against *Campylobacter* is still lacking. To date, many *Campylobacter* immunogenic proteins have been identified and tested as protective antigens in chicken animal models using various delivery vehicles and immunization strategies, but only with partial success. The induction of immune responses (specific intestinal IgA and serum IgG) was documented as a result of immunizations, but the generated reductions of chicken intestinal track colonization by *C. jejuni* were not satisfactory (Wyszyńska et al., 2004; Buckley et al., 2010; Layton et al., 2011; Clark et al., 2012; Neal-McKinney et al., 2012; Theoret et al., 2012) also reviewed in references (de Zoete et al., 2007; Jagusztyn-Krynicka et al., 2009).

The current knowledge indicates that an effective chicken vaccine should induce both a strong and rapid immune response, due to the short life span of broiler chickens. For a short time after hatching, the chicks are protected against *Campylobacter* infection by a high level of maternal antibodies. The mechanism of this protection is not completely clear (Sahin et al., 2001; Sahin et al., 2003; Cawthraw and Newell, 2010). Therefore, birds should be immunized during the first week of life, when the avian immune system is immature. Given this aspect of immunization, we have to deepen our knowledge about *Campylobacter* factors involved in chicken colonization, as well as the pathogen’s interaction with the bird’s immune system (Hermans et al., 2011a). Additionally, with the advances in sequencing technologies, it becomes obvious that the development of a universal effective chicken vaccine against *Campylobacter* is hampered by the *Campylobacter* genome plasticity and antigenic complexity (Friis et al., 2010; Jeon et al., 2010; Gilbreath et al., 2011; Meric et al., 2014). The genetic diversity among *Campylobacter* strains finds reflection in chicken infection biology (Chaloner et al., 2014). Additionally, epidemiological studies indicate that there are multiple *Campylobacter* strains present in broiler flocks at the same time (Newell et al., 2010). So, it is generally thought that only multicomponent subunit vaccines, or using various preparations for the primary vaccination and for the booster, will satisfy immunization requirements.

Recently, the three-dimensional (3D) structures of many antigens have been resolved using mainly two technologies: nuclear magnetic resonance (NMR) spectroscopy and X-ray crystallography, which in combination with bioinformatics strategies allow mapping of the antigen epitopes and can initiate the development of structural vaccinology. This strategy should overcome several limitations in the development of vaccines to protect against pathogens with genetic diversity or antigenic hypervariability (Delany et al., 2014). The first vaccine generated by employing technologies of reverse and structural vaccinology is the 4CMenB multicomponent vaccine against serogroup *Neisseria meningitidis* approved in 2013 by the European Medicine Agency (Serruto et al., 2012).

In this study, we designed and constructed a hybrid CjaA antigen, named the rCjaAD protein, that displays three CjaD peptide epitopes on its surface. Next, the gene encoding rCjaAD was cloned into a *L. lactis* strain in a way that ensured the location of its product to the cell surface. The constructed strain was used for chicken immunization to evaluate the induced immune response and the protective effect of immunization.

## Materials and Methods

### Bacterial Strains, Primers, Plasmids, Media and Growth Conditions

Bacterial strains, plasmids and primers used in this study are listed in **Tables 1** and **2**. The *L. lactis* IL1403 strain used in this study was routinely cultured at 30°C in M17 broth (Oxoid) containing 0.5% (wt/vol) glucose (GM17). When needed, media were supplemented with 5 μg ml^-1^ erythromycin. The *Escherichia coli* strain TG1 was used as a host for the construction of recombinant plasmids. The *E. coli* strain Rosetta (DE3) pLysS was used to overproduce rCjaAD (pUWM1379). *E. coli* strains were grown under standard conditions unless otherwise indicated. When needed, media were supplemented with antibiotics at the following concentrations: 30 μg ml^-1^ kanamycin, 250 μg ml^-1^ erythromycin, or 20 μg ml^-1^ chloramphenicol. *C. jejuni* strain 81–176 was the source of the *cjaA* (*cjj81176_1001, cj0982c*) gene. *C. jejuni* 12/2 strain employed in the protection experiment was a broiler-isolated strain labeled with the pUOA18 plasmid containing a *cat* gene. Previous experiments have shown that the pUOA18 plasmid is stably maintained in *Campylobacter* (Wyszyńska et al., 2004). *C. jejuni* strains were routinely grown at 37°C or 42°C for 16–24 h under microaerobic conditions (5% O_2_, 10% CO_2_, 85% N_2_) on Blood Agar Base No. 2 (BA, Merck, Darmstadt, Germany) plates supplemented with 5% horse blood and “*Campylobacter* Selective Supplement (Blaser-Wang)” (Oxoid, Basingstoke, UK). The medium was supplemented with chloramphenicol (15 μg ml^-1^), if necessary.

**Table 1:** Bacterial strains and plasmids used in this study.

Strain or plasmid	Relevant phenotype(s) or genotype(s)	Source or reference
**Strains**		
*L. lactis* subsp. *lactis* IL1403	Plasmid-free strain	INRA (Chopin et al., 1984)
*E. coli* TG1	*supE thi-1 Δ*(*lac-proAB*) *Δ*(*mcrB-hsdSM*)*5* (*rK^-^ mK^-^*) F’ [*traD36 proAB+ lacI^q^ lacZ_M15*]	Sambrook and Russell, 2001
*E. coli* Rosetta pLysS (DE3)	F^-^ *ompT hsdS*_B_(r_B_^-^ m_B_^-^) *gal dcm* (DE3) pLysSRARE (Cm^R^)	Novagen
*C. jejuni* 81176	Wild type; isolated from a child with bloody diarrhea during an outbreak in Minnesota (USA); pVir, pTet (Tc^R^); Lior 5; Penner 23/26	Korlath et al., 1985
*C. jejuni* 12/2	Wild type; isolated from a chicken; good colonizer; pUOA18 (Cm^R^)	Wyszyńska et al., 2004
**Plasmids**		
pGEM-T Easy	Ap^R^; T vector for cloning PCR products	Promega
pET28a	Km^R^; *lacI*; overexpression vector	Novagen
pBluescript II SK+	Ap^R^; general cloning vector	Stratagene
pIL253	Em^R^, lactococcal cloning vector	Simon and Chopin, 1988
pUWM1000	Em^R^, *L. lactis* /*E. coli* shuttle vector (pIL253 containing ori pBR322)	This study
pUWM1371	*rcjaAD* in Bluescript II SK+	This study
pUWM1379	*6xhis-rcjaAD-6xhis* fusion in pET28a	This study
pUWM1373	Fragment encoding signal sequence of Usp45 (*ss_usp45_*) in pGEM-T easy	This study
pUWM1376	Fragment encoding C-terminal CWA region of YndF (*LPXTG_Y nd_*) in pGEM-T easy	This study
pUWM1381	Fragment encoding signal sequence of Usp45 (*ss_usp45_*) and fragment encoding C-terminal CWA region of YndF (*LPXTG_Y nd_*) in pBluescript II SK	This study
pUWM1384	*ss_usp45__cjaA_LPXTG_Y ndF_* in pBluescript II SK	This study
pUWM1392	*ss_usp45__rcjaAD_LPXTG_Y ndF_* in pUWM1000	This study
pUWM1395	*ss_usp45__cjaA_LPXTG_Y ndF_* in pUWM1000	This study
pUWM1412	*rcjaAD* in pUWM1000	This study


**Table 2:** Oligonucleotides used in this study.

Name of primer	Sequence^∗^	Restriction recognition sites
UspAx	agaggtaccgaattc**TGTTTACCAGCTAGCGCCTA**	KpnI, EcoRI
UspBx	*atggcgcatgccgggcccaa***TGAATTTGTGTCAGCGTAGA**	Sph, ApaI
UspCx	AACGCGTTGCTCGAGACAGATCTAGTCGACCGATATCGGATCCCGCGGCCGCC*ATGGCGCATGCCGGGCCCAA*	MluI, XhoI, BglII, SalI, EcoRV, BamHI, SacII, NcoI, SphI, ApaI
UspDx	TATATCGAT*AACGCGTTGCTCGAGACAGA*	ClaI
YhgE_ClaI_LPXTG	taatcgata**GGCTTGAACTTGGTTGATAA**	ClaI
YhgE_XbaI	agttctagagaattcca**GCCATCATCCCCTCCTAA**	XbaI, EcoRI
YndF_ClaI_LPXTG	ttaatcga**TTGGTAATGCCTCTGGCCAAT**	ClaI
YndF_XbaI_LPXTG	agttctagagaa**TTCCACAACCATTGCCCCTCCTTT**	XbaI, EcoRI
1001_Xho_LPXTG	acctcgagtc**AATTTTTCCACCTTCAATCAC**	XhoI
1001_Bam_LPXTG	acaggatcc**GGAGGAAATTCTGACTC**	BamHI
R_CjaAD_inF	agatccccgggaattctta**AATTTTTCCACCTTCAATCAC**	EcoRI
F_CjaAD_inF	tgattaaatagaattcA**GGAATTGTATGGGAGGAAATTCTGACGAAG**	EcoRI


*^∗^Bold letters indicate C. jejuni or L. lactis nucleotide sequences, and the restriction recognition sites introduced for cloning purposes are underlined. Complementary fragments of primers UspBx + UspCx and UspCx + UspDx are marked with italics. Primers were based on the C. jejuni 81176 or L. lactis IL1403 nucleotide sequences.*

### DNA Manipulations

#### General DNA Manipulations

Standard DNA manipulations were carried out as described earlier by Sambrook and Russell (2001) or according to the manufacturer’s instructions (A&A Biotechnology, Poland). Chromosomal DNAs of *C. jejuni* 81–176 and *L. lactis* used for PCR reactions were isolated using a commercial kit and protocol (A&A Biotechnology, Poland). Polymerase chain reactions (PCRs) were performed with PrimeStar HS DNA Polymerase (TaKaRa) or HotStar HiFidelity Polymerase (Qiagen) under standard conditions. Synthetic oligonucleotide synthesis and DNA sequencing for cloning experiments were performed by Genomed S.A., Warsaw.

#### Construction of Recombinant Plasmids for Recombinant Protein Overexpression

A DNA fragment encoding rCjaAD was synthesized by Genecust and cloned into pBluescript II SK+ digested with PstI and XhoI, generating plasmid pUWM1371. Thereafter, to prepare the rCjaAD overexpression vector, plasmid pUWM1371 was digested with NheI and XhoI restriction enzymes and a 0.9 kb DNA fragment was inserted into pET28a, generating plasmid pUWM1379. Correct construction of the recombinant plasmid was verified by sequencing. Protein production was confirmed by Western blot, using previously obtained rabbit polyclonal anti-CjaD and anti-CjaA sera, and anti–6His serum (Pawelec et al., 2000; Łaniewski et al., 2012). RCjaAD has a 6His tag fused to both the N- and C-termini to allow purification by affinity chromatography.

The construction of the CjaA expression vector (pUWM1146) and CjaD expression vector (pUWM1292) was described previously (Łaniewski et al., 2014; Kobierecka et al., 2015).

#### Construction of the Shuttle Vector pUWM1000

The previously described *L. lactis* plasmid pIL253, based on the pAMβ1 replicon, does not replicate autonomously in *E. coli* (Simon and Chopin, 1988). To circumvent this problem, pIL253 was equipped with an *ori pBR* replicon. A 1.1 kb XbaI-BglII DNA fragment carrying the pBR origin of replication was ligated to pIL253 digested with XbaI and BamHI. The resulting shuttle vector pUWM1000 replicates in both *E. coli* and *L. lactis*.

#### Construction of the Vectors Carrying the Fusion of the *Campylobacter* Genes to the DNA Fragment Encoding the Cell Wall Anchor Region of *L. lactis* YndF

The primers UspAx and UspBx were used to amplify the DNA region encoding the signal sequence of Usp45 (amino acids 1–30) from the chromosome of *L. lactis* IL1403 [PrimeStar HS DNA Polymerase (TaKaRa)]. This region of DNA includes also promoter of the *usp45* gene. The UspBx primer contains a 5′ nucleotide sequence complementary to UspCx primer. PCR product that was purified using a Gel-Out extraction kit (A&A Biotechnology) and the UspCx primer (in equal amounts) was used as a template in a single PCR reaction, using the primers UspAx and UspDx [HiFidelity Polymerase (Qiagen)]. The resulting PCR product was purified and cloned into pGEM-T Easy to generate pUWM1373 (the scheme of pUWM1373 construction is given in **Supplementary Figure S1**). Construction of this recombinant plasmid via a 2-step PCR method allowed the introduction specific restriction enzyme recognition sites, which were useful in cloning procedures.

In order to prepare the translational fusion of *C. jejuni* gene with 3′end of *L. lactis yndF* gene, several steps of genetic manipulation were undertaken. First, the nucleotide sequence encoding the C-terminal CWA region of *yndF* was PCR amplified from the chromosome of *L. lactis* IL1403 with primers YndF_XbaI_LPXTG and YndF_ClaI_LPXTG. The 0.4 kb amplicon was cloned into pGEM-T Easy to generate plasmid pUWM1376. Next, the KpnI-ClaI DNA fragment of pUWM1373, the XbaI-ClaI DNA fragment of pUWM1376 and pBluescript II SK digested with XbaI and KpnI were ligated. The resulting plasmid, designated pUWM1381, contains DNA fragments encoding the signal sequence of the *usp45* gene and a DNA fragment encoding the C-terminal CWA region of YndF in the same transcriptional orientation.

Subsequently, pUWM1381 was used to create the translational fusions of *cjaA* or *rcjaAD* genes with a signal sequence of *usp45* and a nucleotide sequence encoding the C-terminal CWA region of the *yndF* gene. Briefly, *C. jejuni* DNA fragments of 777 bp encoding the *cjaA* gene that lacks its own signal sequence were PCR-amplified using the primer pair 1001_Bam_LPXTG and 1001_Xho_LPXTG from chromosomal DNA. Next, that fragment was cloned into the pUWM1381 recombinant plasmid using BamHI-XhoI restriction enzymes, to generate the pUWM1384. Construction of a pUWM1382 plasmid containing the translational fusion of the *rcjaAD* gene with a nucleotide sequence encoding the C-terminal CWA region of YndF was generated by cloning the 0.94 bp XhoI-SphI DNA fragment of pUWM1371 into pUWM1381 linearized with the same restriction enzymes.

Next, the EcoRI-EcoRI fragments containing translational fusions of *cjaA* or *rcjaAD* genes with a nucleotide sequence encoding the C-terminal CWA region of YndF were transferred into the pUWM1000 *E. coli/L. lactis* shuttle vector, generating pUWM1395 and pUWM1392, respectively. Correct construction of the resulting plasmids was confirmed by restriction analysis and sequencing.

#### Construction of the Vector Encoding *Campylobacter* Antigens of Cytoplasmic Location

In-Fusion^®^ HD Cloning technology was employed to generate pUWM1412, the recombinant plasmid containing the *rcjaAD* gene. The nucleotide sequence encoding the *rcjaAD* gene that lacks its own signal sequence was PCR amplified from the pUWM1371 with primers R_CjaAD_inF and F_CjaAD_inF. The primers used added 15 bp nucleotide sequences to each end of the PCR amplicon that are complementary to the two ends of an EcoRI linearized pUWM1000 vector. The cloning procedure was carried out according to the manufacturer’s instruction (Clonetech). Correct construction of the resulting plasmid was confirmed by restriction analysis and sequencing.

### Transformation of *E. coli* and *Lactococcus lactis*

Recombinant plasmids pUWM1392, pUWM1395 and pUWM1412 were introduced into *L. lactis* IL1403 cells by electroporation as described by Landete et al. (2014). To prepare the competent *Lactococcus* strains, an overnight culture was inoculated 1:50 in GM17 containing 1% glycine and 0.5 M sucrose and incubated at 30°C until an OD_600_ of 0.6 was reached. Bacteria were collected (10,000 g, 10 min, 4°C) and the pellet was washed three times in a washing solution [5 mM KH_2_PO_4_, 2 mM MgCl_2_, 10% glycerol (v/v)] containing 0.5 M sucrose. Bacteria were resuspended 1:100 in the same solution and a volume of 40 μL was electroporated immediately or kept at -70°C for further use. *L. lactis* competent cells were electroporated at 2.5 kV, 200 Ω and 25 μF in 0.4 cm cuvettes using a BioRad GenePulser (BioRad, Life Science Research Products, Hercules, CA, USA). After electroporation, *Lactococcus* cells were resuspended in GM17 broth containing 0.5 M sucrose, 20 mM MgCl_2_ and 2 mM CaCl_2_ and incubated at 30°C for 2 h. Following the incubation, bacteria were plated on M17 containing 0.5 M sucrose supplemented with erythromycin (5 μg ml^-1^). The plates were incubated at 30°C for 2 days.

*Escherichia coli* was transformed as previously described (Hanahan, 1983).

### Recombinant Protein and Polyclonal Antibody Production

#### Overexpression and Purification of CjaA, CjaD and rCjaAD

rCjaAD was overexpressed and purified from *E. coli* Rosetta (DE3) pLysS harboring pUWM1379 by autoinduction, as described by Studier (2005). After 24 h growth, the bacterial culture was centrifuged and the cell pellet was suspended in 50 mM sodium phosphate, 300 mM NaCl, 10 mM imidazole, pH 8.0. Cells were disrupted by sonication. Subsequently, the cell lysate was centrifuged and the resulting supernatant was applied onto a HisTrap column (NGC Chromatography system BioRad). The protein was eluted with an imidazole gradient. Fractions containing rCjaAD were pooled and extensively dialyzed against phosphate buffered saline (PBS) and used for rabbit immunization. Rabbit immunization was carried out according to the ethical standards and with the approval (5666/2014) of the Local Ethics Committee No. 1, Warsaw, Poland.

The anti-rCjaAD rabbit serum was specific and recognized rCjaAD, as verified by Western blot analysis. Overexpression and all purification steps were monitored by SDS-PAGE. Overexpression and purification of CjaA and CjaD were performed using identical method as described previously (Łaniewski et al., 2012; Kobierecka et al., 2015).

#### SDS-PAGE and Western Blotting

SDS-PAGE and Western blotting procedures were done by standard techniques. Blots were developed with nitro blue tetrazolium chloride/5-bromo-4-chloro-3-indolyl phosphate (Sigma–Aldrich) as a substrate, using previously obtained rabbit polyclonal anti-CjaA, anti-CjaD (Pawelec et al., 2000; Łaniewski et al., 2012), or anti-rCjaAD (this work) sera or anti-His antibodies (Sigma–Aldrich) as primary antibodies, and mouse anti-rabbit IgG alkaline phosphatase conjugate (Sigma–Aldrich) or goat anti-mouse IgG alkaline phosphatase conjugate as secondary antibodies.

#### Cellular Localization of the Hybrid Proteins – Immunofluorescence Assay

For the immunofluorescence assay, *L. lactis* IL1403 cells carrying plasmids encoding CjaA or rCjaAD localized in different areas of the bacteria were used. The immunofluorescence assays were carried out as previously reported (Kobierecka et al., 2015).

Rabbit polyclonal anti-CjaA (Pawelec et al., 2000; Łaniewski et al., 2012) or anti-rCjaAD (this work) sera were used as primary antibodies and goat anti-rabbit IgG Alexa Fluor A488 as secondary antibodies. Fluorescence was visualized with a NIKON A1R MP microscope (University of Warsaw).

### Assessment of the Immune Responses and Chicken Protection

#### Growth of Carrier Strain (*Lactococcus lactis* IL1403 Containing *C. jejuni rcjaAD* or *cjaA* genes) for Chicken Immunization

To prepare bacterial suspensions for chicken immunization, an overnight culture of bacteria was diluted 1:50 in fresh pre-warmed GM17 broth and grown at 30°C to an optical density A_600_ = ∼0.4. The cells were sedimented by centrifugation at 8000 × g for 10 min at 4°C, and then suspended in buffered saline with 0.1% gelatin (BSG) to an optical density A_600_ = ∼1 (∼1 × 10^9^ CFU/ml). CFUs were determined by plating serial dilutions of culture on GM17 agar plates supplemented with 5 μg ml^-1^ erythromycin.

#### Immunization and Challenge Regimen

Hy-line chickens were obtained on the day of hatch from a local hatchery. Birds were randomly assigned to experimental groups and housed in an animal facility in separate cages for each group and given water and feed *ad libitum*. Chickens were confirmed to be culture-negative for *Campylobacter* by cloacal swabbing. All animal experiments were carried out according to the ethical standards and with the approval (699/2015) of the Local Ethics Committee No. 1, Warsaw, Poland.

Chickens deprived of food and water for 4 h were orally inoculated with 100 μl of 10^9^ CFU/ml of *L. lactis* carrying pUWM1392 (SP_usp45__rCjaAD_CWA_Y ndF_), pUWM1395 (SP_usp45__CjaA_CWA_Y ndF_) or pUWM1412 (rCjaAD of cytoplasmic location). Booster doses were administrated 8 and 17 days after primary immunization. Following vaccination, chickens were observed for development of diarrhea and other potential adverse side effects. A group of birds inoculated with BSG and *L. lactis* IL1403 were used as a controls. At the 22nd day of life, birds were orally challenged with ∼10^4^ CFU of *C. jejuni* wild-type strain 12/2. At 5 and 9 days post challenge, five birds from each group were euthanized and samples of cecum were collected. Dilutions of the contents were made in PBS and plated onto BA plates supplemented with 5% horse blood, “*Campylobacter* Selective Supplement (Blaser-Wang)” and chloramphenicol (15 μg ml^-1^) for enumeration of *C. jejuni*. Plates were incubated at 37°C for 48 h. Plates that were culture-negative at 48 h were reincubated for an additional 48 h. This procedure permits detection of 10^3^ CFU/g of cecal contents.

To monitor the humoral immune response, three birds from each group were sacrificed on days 7, 14, 21, 27, and 31 post-hatch and samples of serum and gut secretion were collected for the post-mortem examination. On day 1 post hatch, the same number of unvaccinated birds were also euthanized. Blood samples were taken after decapitation. Following centrifugation, sera were collected and stored at -20°C. Samples of gut secretions were collected by intestinal lavage. Secretory IgA antibodies were extracted from lower parts of the intestine with PBS containing 0.05% Tween 20 and soybean trypsin inhibitor (0.1 mg ml^-1^) (dilution 1:10). Samples were shaken for 2 h at 4°C, centrifuged at 20,000 × *g* for 30 min at 4°C, and afterward the supernatant was collected and stored at -20°C.

### Enzyme-Linked Immunosorbent Assay (ELISA)

#### Antigen Preparation

The 6xHis-tagged rCjaAD protein purified as described above was also used as a coating antigen.

#### ELISA Assay

The level of the antibodies against rCjaAD protein in chicken intestinal secretions and blood sera was quantified by ELISA. Briefly, 96-well Maxisorp plates (Nunc, Rochester, NY, USA) were coated with either the purified rCjaAD protein (5 μg per well) or *Campylobacter* membrane proteins (30 μg per well) in PBS and incubated overnight at 4°C. Then, plates were blocked for 1 h at 37°C with PBS containing 0.1% Tween 20 (Sigma–Aldrich) and 1% BSA (bovine serum albumin), washed three times with PBS containing 0.1% Tween 20 (Sigma–Aldrich) and incubated for 1 h at room temperature with the diluted sera (1:256) or intestinal secretion samples (1:10). Goat anti-chicken IgA horseradish peroxidase conjugate (Thermo Fisher, Scientific) was employed to detect chicken IgA that bound to *Campylobacter* antigens. The plates were developed with 3,3′,5,5′-tetramethylbenzidine (Sigma–Aldrich), according to the manufacturer’s directions. The reaction was stopped with 3M H_2_SO_4_ and optical density was determined at *A* 490 using an ELISA reader (Tekan). The level of specific serum IgG was measured using rabbit anti-chicken IgY (whole molecule) alkaline phosphatase conjugate (Sigma–Aldrich). The reaction was run with *p*-nitrophenyl phosphate (1 mg ml^-1^) as substrate and was stopped after 30 min incubation (room temperature) with 3N sodium hydroxide. Optical density was determined at 405 nm using an ELISA reader (Tekan). Each sample was analyzed in triplicate.

### Bioinformatics Analyses

#### Protein Structure Prediction

Secondary structure prediction and tertiary fold-recognition (FR) were carried out via the GeneSilico metaserver gateway (Kurowski and Bujnicki, 2003). Solvent accessibility for the individual residues was predicted with SABLE (Adamczak et al., 2005), ACCPRO2 (Cheng et al., 2005a), and JNET (Cuff and Barton, 2000). Disordered residues were predicted from consensus of results generated with disprot (Dunker et al., 2002), DISpro (Cheng et al., 2005b), DisEMBL (Linding et al., 2003), RONN (Yang et al., 2005), and disopred (Ward et al., 2004) methods. Secondary structure was predicted using a consensus of PSIPRED (Jones, 1999b), PROFsec (Rost et al., 2004), PROF (Ouali and King, 2000), SABLE (Adamczak et al., 2005), JNET (Cuff and Barton, 2000), JUFO (Meiler and Baker, 2003), PORTER (Pollastri and McLysaght, 2005), SSPRO2 (Cheng et al., 2005a), and SAM-T02 (Karplus et al., 2003). In all these cases results generated by independent methods were compared and a consensus result was retrieved as a most probable solution. The FR analysis (attempt to match the query sequence to known protein structures) was carried out using PDBBLAST, HHSEARCH (Soding, 2005), FFAS03 (Jaroszewski et al., 2005), FORTE (Tomii and Akiyama, 2004), SAM-T02 (Karplus et al., 2003), 3DPSSM (Kelley et al., 2000), INBGU (Fischer, 2000), FUGUE (Shi et al., 2001), mGENTHREADER (Jones, 1999a), and SPARKS (Zhou and Zhou, 2004). Target-template alignments reported by these methods were compared, evaluated, and ranked by the PCONS server (Lundstrom et al., 2001) to identify the preferred modeling templates and the consensus alignment. The alignments between the amino acid sequences of CjaA and CjaD and the structures of the best templates identified by FR were used to carry out homology modeling using Modeler (Sali and Blundell, 1993). For the evaluation of models, a MetaMQAP method was used. (Pawlowski et al., 2008), which allows predicting the deviation of individual amino acid residues in the model from their counterparts in the native structure.

#### Prediction of Epitopes

Epitopes were predicted from sequence using the following methods: Chou and Fasman Beta-Turn Prediction (Chou and Fasman, 1978), Emini Surface Accessibility Prediction (Emini et al., 1985), Karplus and Schulz Flexibility Prediction (Karplus and Schulz, 1985), Kolaskar and Tongaonkar Antigenicity (Kolaskar and Tongaonkar, 1990), Parker Hydrophilicity Prediction (Parker et al., 1986), and Bepipred Linear Epitope Prediction (Larsen et al., 2006). All results were compared and consensus predictions were mapped on homology models of CjaA and CjaD proteins. We considered consensus fragments that were located in loops exposed to the solvent to be the most probable epitopes.

### Statistical Analyses

Statistical analyses of colonization results were performed using GraphPad Prism 6 (GraphPad Software, Inc., San Diego, CA, USA). The significance of differences between the obtained values was appraised using one-way analysis of variance (ANOVA) followed by the *post hoc* Tukey test. Statistical analyses of ELISA test were assessed using the Kruskal–Wallis test followed by Dunn’s multiple-comparison *post hoc* test. Statistical analyses were performed using STATISTICA 10PL software (StatSoft, USA). Any *p*-values <0.05 were considered significant.

## Results

### Protein Structure Prediction

#### CjaA

As a starting point for a structural model of CjaA, a protein FR analysis of the CjaA amino acid sequence was performed. FR methods attempt to identify the most appropriate modeling templates for a query sequence and return a set of alternative alignments to proteins of known structure. Most FR methods available in the MetaServer consistently reported the structure of the crystal structure of the cysteine-binding protein from *C. jejuni* (PDB code: 1xt8) as the potentially best template for modeling the CjaA protein (top matches to 1xt8, with all methods). However, the FR analyses showed no statistically significant similarity to any structurally characterized template for the N terminal part of CjaA (residues 1–25). A model of the central domain (starting from residue 26) was constructed using the ‘FRankenstein’s Monster’ protocol (Kosinski et al., 2005), and as expected from comparative modeling, the CjaA revealed features of its templates. The prediction of the quality of the final model yields good scores with MQAP methods. The program MetaMQAP predicted that the whole model exhibits a root mean square deviation from the true structure on the order of 5.2 Å, and a predicted GDT_TS score of 41.667. These results suggest that our model of CjaA is sufficiently reliable as a framework to interpret sequence–structure-function relationships in the CjaA, such as analysis of putative epitopes.

#### CjaD

As a starting point for a structural model of CjaD, a protein FR analysis of the CjaD amino acid sequence was performed. Most FR methods available in the MetaServer reported two structures: the crystal structure of TolB/Pal complex and the solution structure of peptidoglycan associated lipoprotein from *Haemophilus influenzae* bound to UDP-*N*-acetylmuramoyl-L-alanyl-D-glutamyl-meso-2,6-diaminopimeloyl-D-alanyl-D-alanine (PDB codes: 2hqs and 2aiz) as the potentially best templates for modeling of the CjaD protein. However, the residues 1–50 were identified as intrinsically disordered by all methods available in the Metaserver. A model of the central domain (starting from residue 50) was constructed using the ‘FRankenstein’s Monster’ protocol (Kosinski et al., 2005) based on two template structures. The prediction of the quality of the final model yields good scores with MQAP methods. The program MetaMQAP predicted that the whole model exhibits a root mean square deviation from the true structure on the order of 6.7 Å, and a predicted GDT_TS score of 34.39. These results suggest that our model of CjaD is sufficiently reliable as a framework to interpret sequence–structure-function relationships in the CjaD, such as analysis of its putative epitopes.

#### Prediction of Epitopes

**FIGURE 1:**
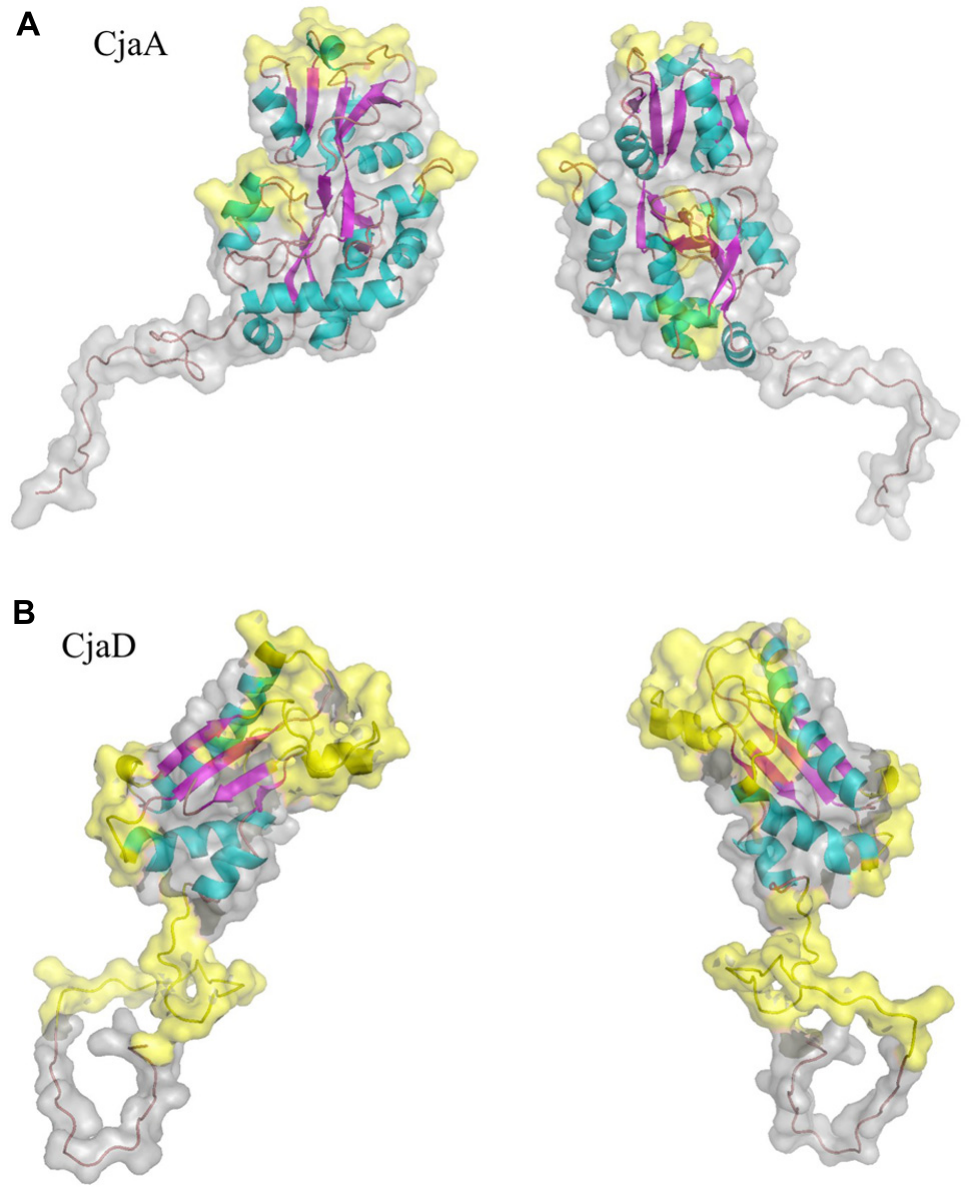
**Ribbon and surface diagram of the full-length protein model **(A)** CjaA, **(B)** CjaD.** Potential peptide epitopes are shown in yellow.

As a starting point to identify epitopes in CjaA amino acid sequence we performed epitope identification based on amino acid sequence using five methods (references as in Materials and Methods). Then, we mapped amino acid residues identified by all five methods onto the comparative models of CjaA and CjaD built in this work. As putative epitopes, we defined only those from predicted regions of the amino acid sequences that were located on the protein surfaces and visibly exposed to the solvent (**Figure 1**). The following CjaA fragments were selected as the most probable epitopes: 55–60, 79–82, 111–118, 136–149, 167–171, and 263–268. In the case of CjaD, the 87–91, 99–108, and 139–158 amino acid fragments were indicated as the most probable peptide epitopes.

**FIGURE 2:**
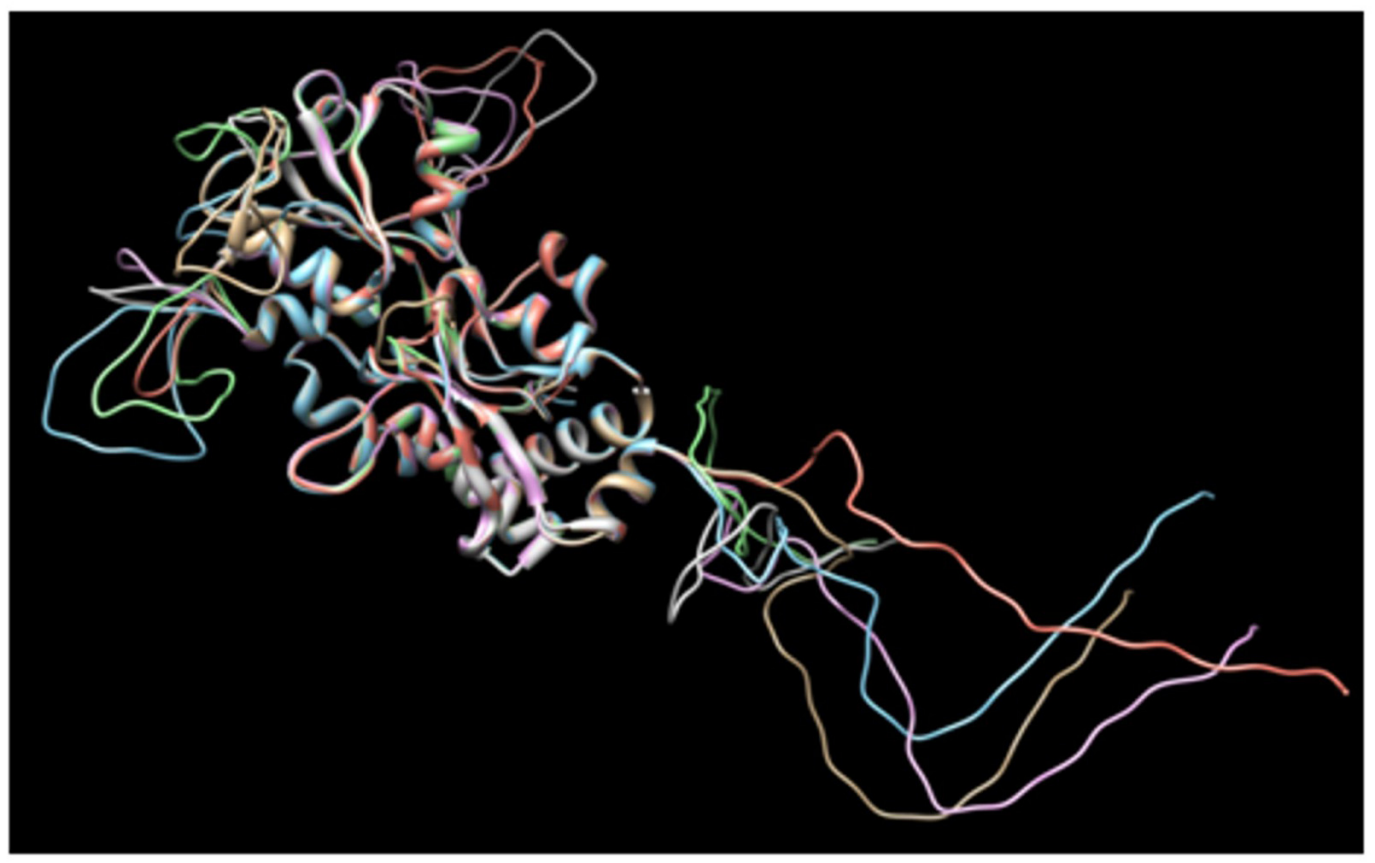
**Dimensional structures of each of the proposed amino acid sequences of CjaA with the three insertions of CjaD peptide epitopes**.

Additionally, based on the aforementioned predictions, six alternative amino acid sequences of CjaA with inserted CjaD epitopes were designed. For this purpose, first we selected five loops in CjaA model that were exposed to the solvent (25–26, 88–89, 189–190, 207–208, 218–219) and inserted three CjaD epitopes (EVSGV, DEWGTDEYN, GETNPVCTEKTKACDAQNRR) in all possible combinations (**Supplementary Figure S2**). Thus, we obtained six alternative amino acid sequences with three CjaD epitopes each. Finally, we built homology models for all six hybrid amino acid sequences (rCjaAD). The obtained models confirmed that cores of the proteins are unchanged whereas epitopes are inserted into loops exposed to the solvent (**Figure 2**).

### Construction of CjaA Protein Displaying CjaD Peptide Epitopes on its Surface; Analysis of its Antigenicity and Immunogenicity

**FIGURE 3:**
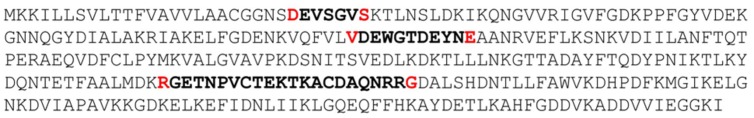
**The amino acid sequence of rCjaAD protein.** Amino acids between which peptide epitopes of CjaD protein (indicated in bold) were inserted are indicated in red.

The structure based approach combined with the identification of the CjaA and CjaD epitopes allowed us to construct a CjaA antigen that presents CjaD epitopes on its surface. Three epitope amino acid sequences (EVSGV, DEWGTDEYN, GETNPVCTEKTKACDAQNRR) from CjaD were inserted at positions 25–26, 88–89, 189–190 of CjaA amino acid sequence (**Figure 3**). The DNA fragment encoding hybrid protein lacking the CjaA signal sequence (rCjaAD) was synthesized by Genecust and cloned into pBluescript II SK+ (see Materials and Methods). Next, it was recloned into pET28a and introduced into *E. coli* Rosetta (DE3) pLysS. rCjaAD was overproduced and purified by affinity chromatography.

**FIGURE 4:**
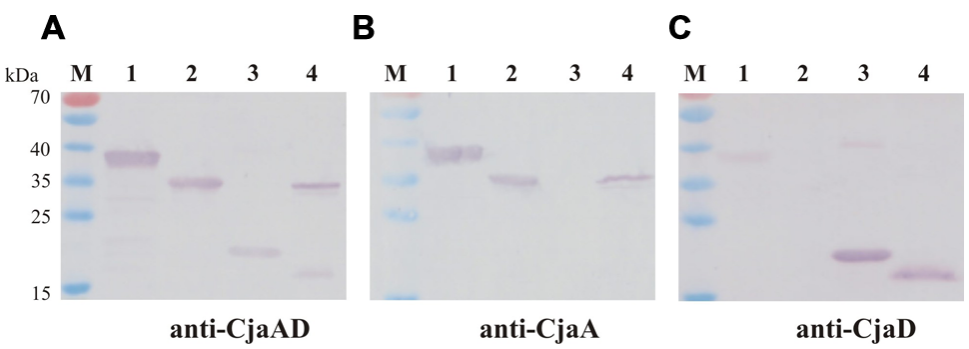
**Western blot analysis of the specificity of rCjaAD.** Protein extracts were separated by 12% SDS-PAGE under reducing conditions and probed with: **(A)** anti-rCjaAD antibodies, **(B)** anti-CjaA antibodies, **(C)** anti-CjaD antibodies. Lanes: 1 – purified protein 6xHis-rCjaAD-6xHis, 2 – purified protein 6xHis-CjaA; 3 – purified protein 6xHis-CjaD; 4 – *C. jejuni* cell lysate, M - protein molecular-weight marker.

Next we checked the specificity of rCjaAD by Western blot experiments. We found that rCjaAD protein reacts with specific rabbit anti-CjaA, as well as with specific rabbit anti-CjaD sera (**Figures 4B,C**, lane 1). As the hybrid protein was constructed with the aim of vaccination, we also investigated its immunogenicity. We found that the specific serum obtained by rabbit immunization with rCjaAD reacted strongly with both the native CjaA and the native CjaD produced by a wild type *C. jejuni* strain (**Figure 4A**, lane 4).

### Construction of the *L. lactis* Strains Expressing *C. jejuni* Genes

We constructed two plasmids expressing a fusion of the *C. jejuni* CjaA or rCjaAD with the cell wall anchor region of YndF (CWA-YndF): **LPETG**DKEQGMKKITLFGSFLLILGSLVLFIRFRKVD). YndF (NP_267457) is a substrate for sortase SrtA. Two mucin-binding domains were identified in this protein, which suggests its possible function in adhesion to epithelial cells or possibly other cells (Dieye et al., 2010).

All genetic manipulations were performed in *E. coli*. To begin, we constructed a shuttle vector pUWM1000 able to replicate in *E. coli* and *L. lactis* strains. The shuttle vector is a derivative of a patented *L. lactis* plasmid carrying erythromycin resistance (pIL253), in which the ori pBR was introduced in order to ensure its replication in *E. coli* cells. The plasmid was introduced into *E. coli* by chemotransformation and *L. lactis* by electroporation.

**FIGURE 5:**
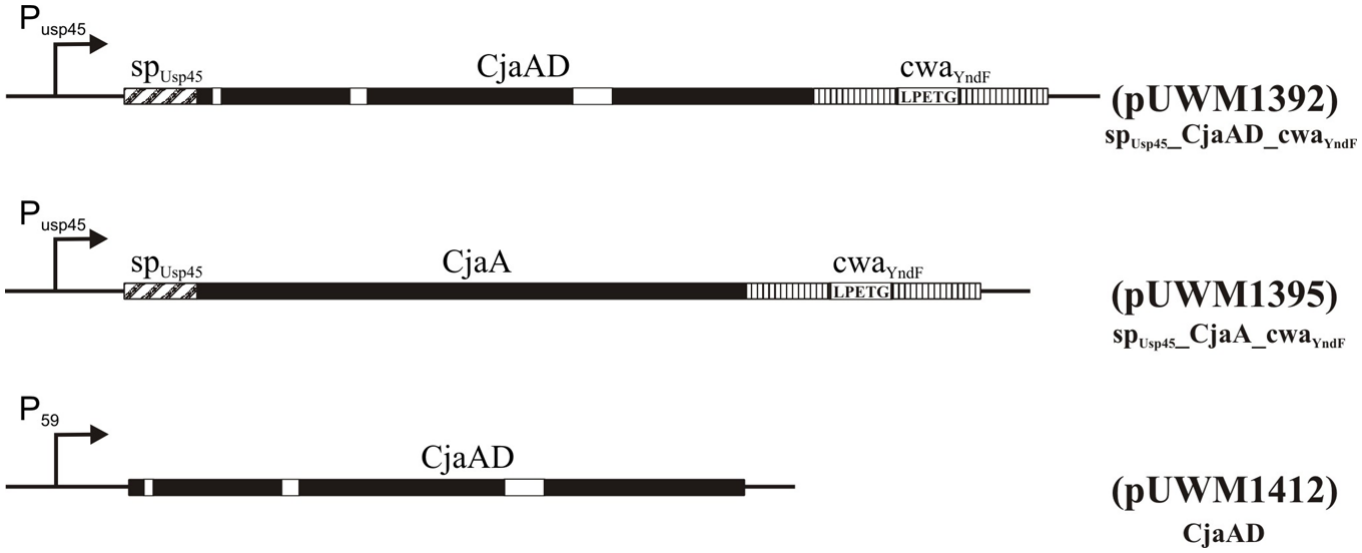
**Schematic representation of the hybrid proteins.** P_59_ and P_Usp45_ are strong, constitutive lactococcal promoters (van der Vossen et al., 1987), *sp* refers to the signal peptide of Usp45 (van Asseldonk et al., 1990) and *cwa* to the cell wall anchor region of the lactococcal protein YndF. Epitopes of CjaD proteins are marked with white rectangles. The corresponding plasmids are indicated in parentheses.

Next, specific recombinant plasmids to generate fusions of *C. jejuni* and *L. lactis* genes were created. First, the promoter and DNA fragment coding for a signal peptide (SP) of Usp45 *L. lactis* protein (a 45 kDa secreted protein of unknown function) was introduced into pGEM-Easy to allow secretion of produced antigens. At the same time, the PCR-amplified DNA fragment encoding the CWA region of YndF was inserted into pGEM-T Easy. The resulting plasmids (pUWM1373 and pUWM1376, respectively) were the source of fragments, which were connected in the same transcriptional orientation in pBluescript II SK in the next step (pUWM1381). There are different nucleotide sequences recognized by restriction enzymes located at the junction between the 3′ end of the DNA region encoding the SP_Usp45_ signal sequence and the 5′ end of *L. lactis* DNA encoding the CWA region of YndF. This strategy facilitates cloning of *C. jejuni* genes. The last stage of the construction work included the insertion of two *C. jejuni* genes (encoding CjaA and rCjaAD, respectively) in such a way that both are expressed from the Usp45 promoter. The proteins lack the CjaA signal sequence but are equipped with the Usp45 signal sequence and are fused to the C-terminus of YndF. The details of construction are shown in **Figure 5** and **Supplementary Figure S3**.

Finally, the DNA fragments of pUWM1384 or pUWM1382 encoding CjaA or rCjaAD fused to C-terminus of YndF were transferred into the pUWM1000 shuttle vector to generate pUWM1395 and pUWM1392, respectively. The correctness of constructed recombinant plasmids was verified by sequencing at every step of work. pUWM1392 and pUWM1395 were introduced into *L. lactis* by electrotransformation. Next, we confirmed the production of CjaA and rCjaAD by *L. lactis* by Western blotting experiments. We found that *L. lactis* contained plasmids harboring the *rcjaAD* or *cjaA* genes expressed proteins with approximate molecular masses of ∼53 or ∼50 kDa, respectively. Molecular masses of the proteins reacting with specific rabbit sera against CjaA or rCjaAD were consistent with the calculated sizes of the CjaA or rCjaAD fusions with CWA region of YndF (**Supplementary Figure S4**, lines 1 and 2).

To confirm the impact of the C-terminus of YndF on protein location and subsequently on the efficacy of vaccination, we also constructed two *L. lactis* strains harboring the recombinant plasmid that encodes *Campylobacter* rCjaAD antigen with a cytoplasmic localization (pUWM1492). Details of its construction are described in the methods section. The protein produced by this strain, which reacts with anti-rCjaAD serum, has the expected molecular mass (**Supplementary Figure S4**, line 3).

### Localization of CjaA and rCjaAD Proteins

**FIGURE 6:**
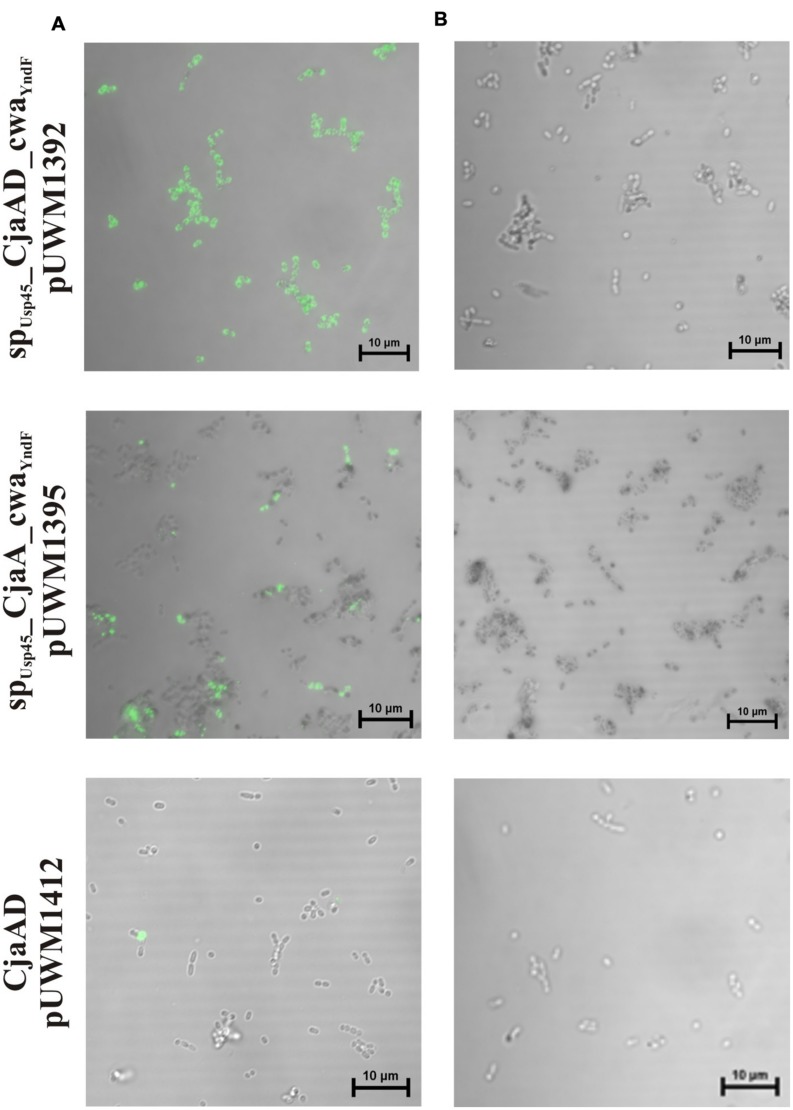
**Localization of SP_Usp45__rCjaAD_CWA_Y ndF_, SP_Usp45__CjaA_CWA_Y ndF_ and CjaA proteins on *L. lactis* IL1403 cells.** Microscopic observations of the *L. lactis* strains expressing the fusion genes: *sp_Usp45__rcjaAD_cwa_Y ndF_*, *sp_Usp45__cjaA_cwa_Y ndF_* and *rcjaAD*. **(A)** Shows bacterial cells whose fusion proteins were visualized with anti-CjaA or anti-rCjaAD antibodies that were further detected with goat anti-rabbit IgG Alexa Fluor A488. **(B)** Primary antibodies were omitted (negative controls). Fluorescence was visualized with a NIKON A1R MP microscope. The bar represents 10 μm.

We next investigated the localization of CjaA and rCjaAD by immunofluorescence assay. We found that the *L. lactis* IL1403 strain bearing the fusion SP_Usp45__rCjaAD_CWA_Y ndF_ displayed strong fluorescence. Fluorescence was also observed for SP_Usp45__CjaA_CWA_Y ndF_ protein, though not as intense as the SP_Usp45__rCjaAD_CWA_Y ndF_ protein. In contrast, no fluorescence was observed in cells of *L. lactis* strain harboring a recombinant plasmid encoding *Campylobacter* rCjaAD antigen with a cytoplasmic location. The results are given in **Figure 6**.

### Chicken Immunization with *L. lactis* Producing Surface Exposed Hybrid Protein rCjaAD

Many Lactic Acid Bacteria (LAB) have received Generally Regarded as Safe (GRAS) status, and some of them are recognized as probiotics (Fontana et al., 2013; Wyszyńska et al., 2015). This makes LAB strains such as *L. lactis* suitable as potential vectors for chicken vaccination against *Campylobacter*. Our constructed *L. lactis* strain, expressing surface exposed rCjaAD, was used for chicken immunization in order to evaluate how well it provided protection against *Campylobacter* infection and to assess the induced immune responses. Additionally, to assess the role of protein localization and to evaluate the impact of a hybrid protein containing epitopes derived from two immunogenic proteins, two additional *L. lactis* strains were included in the experiment. One (*L. lactis* /pUWM1412) produces cytoplasm-localized rCjaAD, and the second produces surface-localized CjaA (*L. lactis* /pUWM1395). The scheme of the experiments is depicted in **Table 3**.

**Table 3:** Scheme of immune response and protection experiments.

Day of life	1	7	8	14	17	21	22	27	31
Immunization with *Lactococcus*	+		+		+				
Collection of blood and gut secretion samples for immune response analyses	+	+		+		+		+	+
Challenge with *Campylobacter*							+		
Cecum isolation for *Campylobacter* enumeration								+	+


*Note: + indicates when the procedures occurred.*

Briefly, three groups of 1-day old chickens (19 per each group) were orally immunized with *L. lactis* strains expressing *C. jejuni* antigens (details are given in Materials and Methods). Chickens were boosted with the same doses of the same strains at 8 and 17 days post-hatch. Two groups of birds, one inoculated with BSG and the second inoculated with *L. lactis* IL1403 were used as a controls. The rationale behind this schedule of immunization is the fact that the immune system of chickens remains immature for the first 2 weeks of life (Mast and Goddeeris, 1999; Bar-Shira et al., 2003). Additionally, maternal antibodies, mainly directed against outer-membrane proteins, may restrict the induction of an immune response.

### Serum and Intestinal Antibody Responses

**FIGURE 7:**
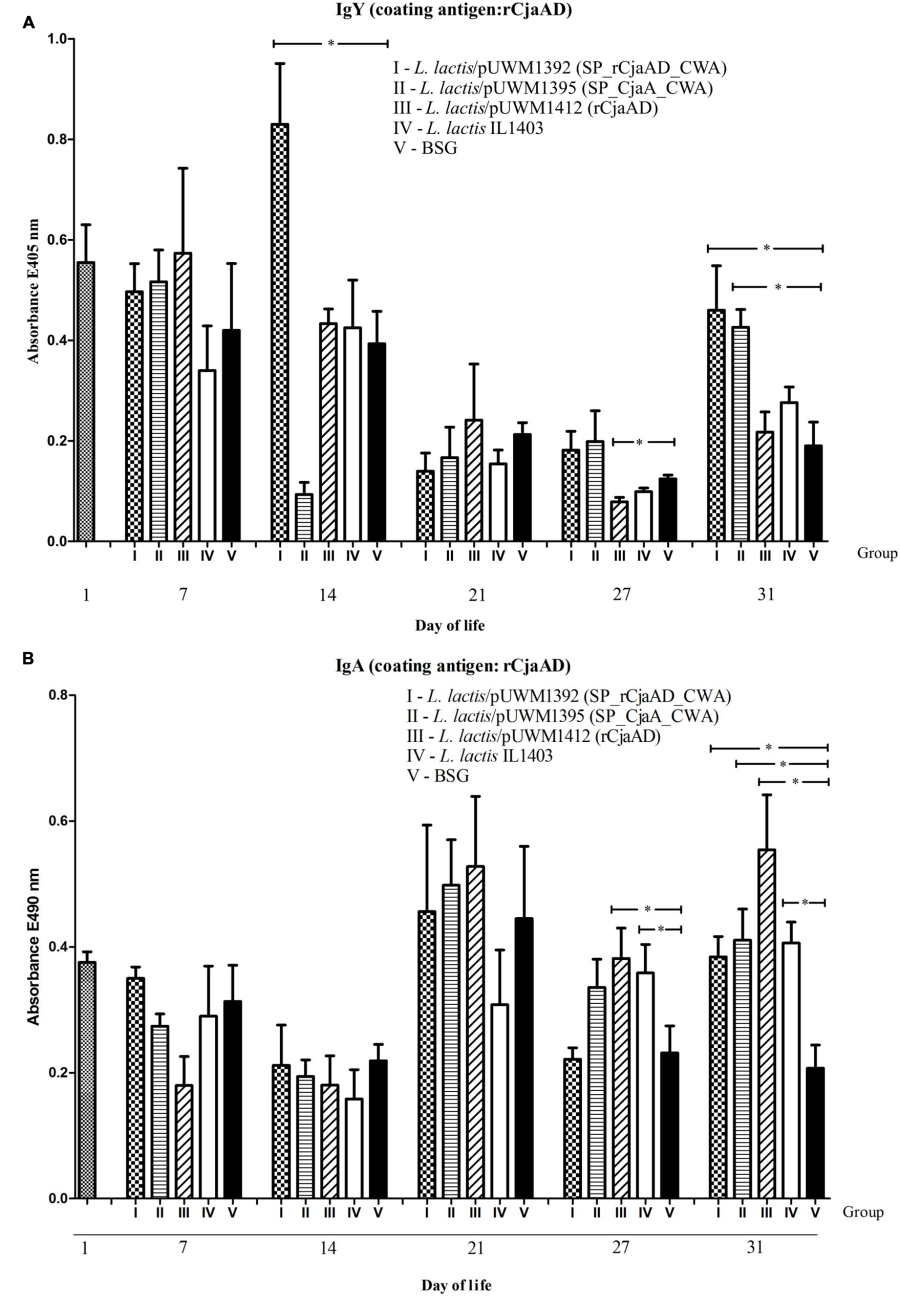
**Immune responses of chickens vaccinated with *L. lactis* strains (*L. lactis*/pUWM1392, *L. lactis*/pUWM1395 and *L. lactis*/pUWM1412).** Levels of serum IgY **(A)** and mucosal sIgA **(B)** antibodies specifically recognizing CjaA or rCjaAD antigens were determined by ELISA. Chickens were given three doses of the vaccine strains at 1, 8, and 17 days post-hatch. Birds were infected with *C. jejuni* at 22 day of life. Control birds were given *L. lactis* IL1403 strain (open bars) or BSG (black bars). Serum and intestinal samples were collected at the specified days of chicken life. Purified rCjaAD protein was used as a coating antigen. Serum samples were diluted 1:256 and intestinal secretion samples 1:10. Absorbance values represent a mean of 5 birds ±SD per time interval. A statistical analysis was carried out using the Kruskal–Wallis test followed by Dunn’s multiple-comparison *post hoc* test. An asterisk indicates significant difference (*p* < 0.05) between groups and control group (group V).

To investigate the immunogenicity of the surface-exposed rCjaAD hybrid protein delivered by *L. lactis*, serum IgYs and mucosal IgAs were measured by ELISA using rCjaAD as coating antigen. The kinetics of the induction of two kinds of antibodies (specific IgYs and specific IgAs) varies significantly (**Figures 7A,B**). Systemic IgY responses to *Campylobacter* antigens were observed at days 7 and 14 (after the first and the second dose of vaccine), decreased at days 21 and 27, and finally increased at day 31 when the experiment was terminated (**Figure 7A**). The high level of specific IgYs observed during the first 2 weeks of chicken life represents maternal antibodies. The vaccinated groups (with one exception) had higher levels of specific IgYs on days 7 and 14 than the group vaccinated with carrier strain. These results indicate that vaccination may support the protective activity of maternal antibodies. The extremely high level of specific IgG induced by *L. lactis*/pUWM1392 (surface exposed rCjaAD) and low level induced by *L. lactis*/pUWM1395 (surface exposed CjaA) observed at day 14 is not clear. The anti-rCjaAD IgY response at day 31 elicited by *L. lactis* harboring *Campylobacter* antigens is significantly higher than the control group. The highest titer of the anti-rCjaAD antibodies was detected after immunization with the *L. lactis* that presented hybrid protein on its surface. Generally, mucosal IgAs are regarded as the first line of immune defense against many pathogens. Anti-rCjaAD mucosal IgA titers increased in all immunized group after the second booster (day 21). In the case of specific anti-rCjaAD mucosal IgAs, no correlation was noticed between the level of the induced immune response and antigen localization. Anti-rCjaAD IgA responses in the three groups immunized with *L. lactis* strains were similar, both in kinetics and titer at all points of the experiment (**Figure 7B**).

### Protection Analysis

To determine whether the rCjaAD delivered by *L. lactis* provides protection against *Campylobacter* infection, immunized chickens were challenged orally with 8 × 10^4^ bacterial cells of a broiler-isolated *C. jejuni* strain 5 days after the second booster. The *C. jejuni* strain used for the challenge experiment was labeled with the pUOA18 plasmid containing a *cat* gene. Protection was assessed at 5 and 9 days after the challenge by plating to determine the level of bird’s caeca colonization by wild type *Campylobacter*.

**FIGURE 8:**
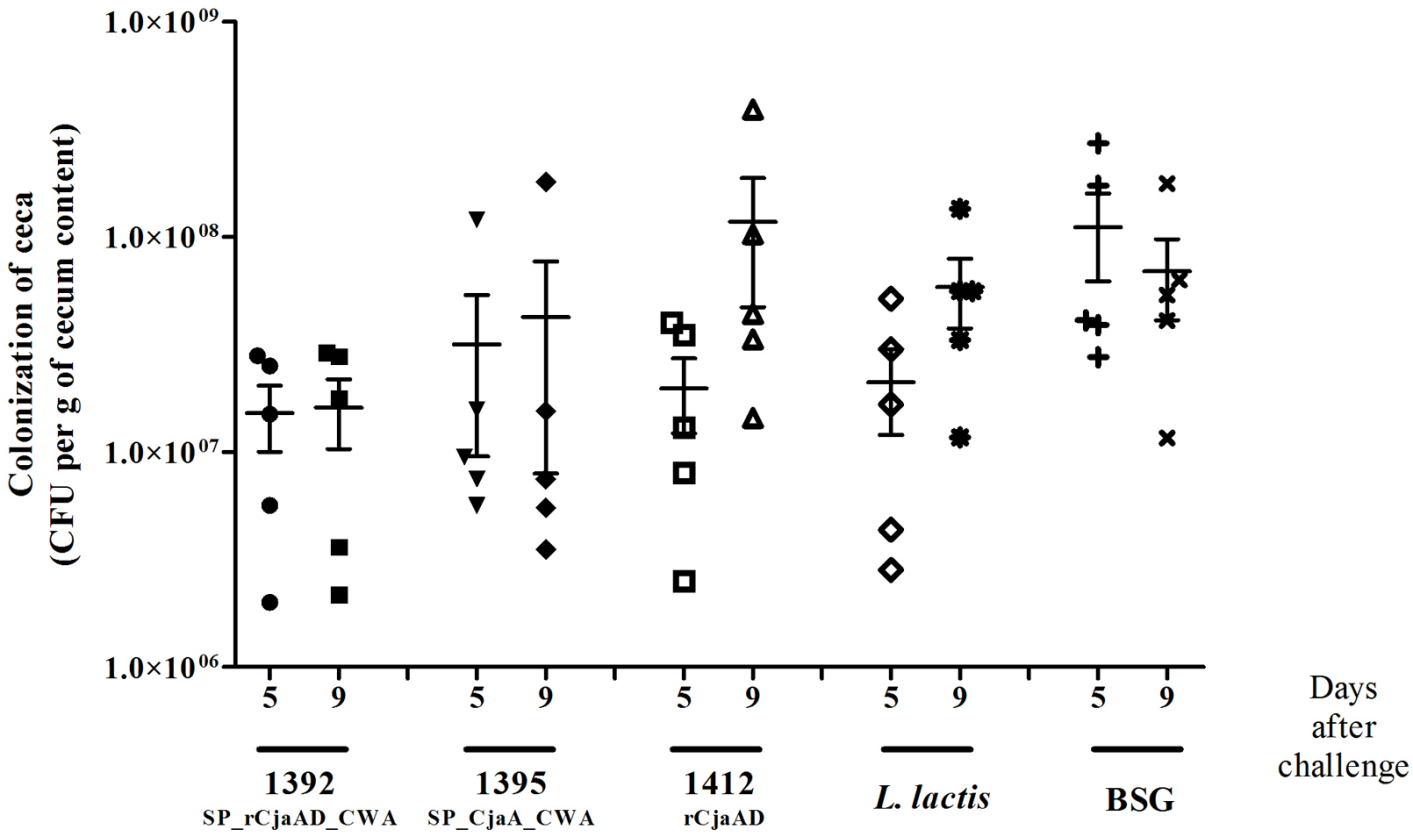
**Colonization of chickens vaccinated with *L. lactis* strains (*L. lactis*/pUWM1392, *L. lactis*/pUWM1395 and *L. lactis*/pUWM1412) and then given a *C. jejuni* challenge.** Chickens were given three doses of the vaccine strains at 1, 8, and 17 days after hatch and challenged with *C. jejuni* 12/2/pUOA13 5 days later. Control birds were given BSG. Viable *C. jejuni* cells were recovered from the ceca of chickens at specified days after challenge. Bacterial recoveries represent colonization levels of 5 birds per time interval. The geometric mean for each group is denoted by bars. No significant differences (*p* < 0.05) between groups were seen.

There was a noticeable, though not statistically significant, reduction in the CFUs (colony forming units) of *C. jejuni* in the cecal contents of birds immunized with *L. lactis* that produced surface-exposed rCjaAD (pUWM1392), as compared to control group (**Figure 8**). The mean CFU/gram of cecal content observed in this group was about 1 × 10^7^ whereas the mean level of colonization in the control group was 1 × 10^8^ CFU/gram. In the control group receiving *L. lactis* vector strain, no reduction in the colonization level was noticed, as compared to group receiving BSG, especially 9 days after the challenge. It should be noted that among three vaccinated groups, the group immunized with *L. lactis* producing surface-located rCjaAD displayed the lowest range in the level of colonization. Additionally in this group, the immunization of two out of six birds resulted in reduction of cecal *C. jejuni* by about 2 log_10_ units compared to birds receiving BSG at 9 days after challenge. Based on the mean level of colonization, vaccination with *L. lactis*/pUWM1395 (surface-exposed CjaA) does not result in protection against *C. jejuni* colonization. However, in this case the highest range of colonization levels was observed between individual chickens. While vaccination with *L. lactis*/pUWM1392 producing surface-located rCjaAD resulted in reduction of *C. jejuni* colonization at 5 and 9 days post-challenge, the reduction observed after immunization with an *L. lactis* strain producing the same antigen, but cytoplasmically located, is noted only for a short time. At day 9 post-challenge, the mean level of colonization was even slightly higher compared to the non-vaccination group. However, this group also had significant differences in the level of colonization between individual birds.

## Discussion

*Campylobacter* sp. infection remains the leading cause of human food-borne gastroenteritis in industrialized countries. The occurrence of high loads of *Campylobacter* cells in the chicken digestive tract is still prevalent in broiler flocks (EFSA and ECDC, 2015). So there is an urgent need to control chicken contamination by *Campylobacter*. Many interventions aimed at lowering the level of chicken-carcass contamination during the poultry production cycle have recently been proposed and tested. However, the currently available interventions are of limited effectiveness or difficult to sustain (Hermans et al., 2011b; Josefsen et al., 2015). Thus, the market needs an effective anti-*Campylobacter* chicken vaccine. Recent progress has been made to understand the complex *Campylobacter* biology during chick colonization, as well as improvements in the technologies used for identifying the immunodominant proteins (Hermans et al., 2011a; Hoppe et al., 2014; Hu et al., 2014); both of these facilitate the selection of antigens for immunization.

Based on the results of chicken vaccination experiments presented by us and others (Wyszyńska et al., 2004; Buckley et al., 2010; Layton et al., 2011), and also on known facts about *Campylobacter* physiology and genomics, we decided to use two conserved, extracytoplasmic and highly immunogenic proteins, CjaA and CjaD (Konkel et al., 1996; Pawelec et al., 1997; Pawelec et al., 2000), for the experiments in this paper. CjaA is a periplasmic, cysteine binding protein (Muller et al., 2005), and CjaD is a peptidoglycan-associated essential protein (PAL) responsible for the integrity of bacterial cell wall (Godlewska et al., 2009). Additionally, CjaA was found among the proteins recognized by maternal antibodies that protect young chicks against *Campylobacter* infection (Shoaf-Sweeney et al., 2008). Analysis of the genomes of members of the *Campylobacter* genus, species other than C. *jejuni*, indicate CjaA as a core-virulence factor and potential candidate for subunit vaccine development (Ali et al., 2012). Given that several *Campylobacter* species have recently been recognized as emerging animal or human pathogens, this particular characteristic of CjaA is significant (Kaakoush et al., 2015). To facilitate antigen production by the vector strain and to overcome, at least partially, the problem created by the various *Campylobacter* genotypes, we constructed a hybrid protein: a CjaA that presents CjaD epitopes on its surface. Detailed bioinformatics analysis allowed us to determine the CjaD epitopes and specify the appropriate places to introduce them into the CjaA amino acid sequence. Structural modeling verified the construction of the hybrid protein. It showed that the CjaA structure was not disturbed and that the selected CjaD epitopes were present on the CjaA surface. The immunogenicity of rCjaAD was documented by rabbit immunization; the rabbit serum obtained after immunization with rCjaAD reacted with CjaA or CjaD present in a wild type *Campylobacter* strain. A similar approach has recently been presented by Konkel et al. (1996) who showed that intramuscular immunization with a subunit vaccine that consists of epitopes from three surface-exposed colonization proteins (CadF-FlaA-FlpA), combined with adjuvant, gave a significant reduction of *Campylobacter* colonization (Neal-McKinney et al., 2014). However, due to the biology of *Campylobacter* infection it is generally considered that oral antigen administration will be the most effective way to immunize chickens. As shown by Theoret et al. (2012) the mode of antigen delivery is sometimes crucial for immunization. They noticed that subcutaneous vaccination with purified rDps (DNA binding protein from starved cells) did not reduce colonization of chickens by *Campylobacter*, whereas the same protein delivered by an attenuated *Salmonella* appeared to be effective (Theoret et al., 2012). The importance of humoral immune response in preventing chicken colonization by *Campylobacter* has been recently documented by Hermans et al. (2014). They showed that passive immunization of 6 days old chicks with IgY obtained from egg yolks of hens immunized with *C. jejuni* lysates or fraction of *C. jejuni* hydrophobic proteins resulted not only in reduction their colonization by homologous *C. jejuni* strain but also reduce the pathogen transmission to not infected birds (Hermans et al., 2014). More and more LAB, mainly members of the *Lactococcus* and *Lactobacillus* genera, have been tested as vehicles for the delivery of heterologous bacterial or viral antigens into animal mucosal immune systems (Marelli et al., 2011; Villena et al., 2011; Kajikawa et al., 2012; Fontana et al., 2013; Li et al., 2014). In this work, we employed *L. lactis* because genetic engineering tools for this bacterium have been worked out in detail (Fontana et al., 2013). To the best of our knowledge, our work is the first attempt to use an LAB strain as a *Campylobacter* antigen delivery vehicle for chicken immunization. Although we observed a positive result from immunization, the reduction of chicken digestive tract colonization by *Campylobacter* after vaccination was not significant and was lower than that described previously by us and others (Wyszyńska et al., 2004; Layton et al., 2011). The median reduction in *C. jejuni* cecal contents observed after vaccination with surface-exposed hybrid protein was 1 log_10_ However, it should be noted than there was about a 2 log_10_ reduction in the level of colonization for four out of 10 birds. The main drawback of the *L. lactis* delivery vehicle is the lack of long-lasting colonization in the chick digestive tract. Thus, we postulate that it may be necessary to optimize the number of vaccine doses to improve the effectiveness of vaccination. Alternatively, using *Lactobacillus* sp. that colonize bird intestines should result in more efficient induction of the immune response (Wyszyńska et al., 2015).

The significant differences among published results of chicken vaccination against *Campylobacter* are apparent. The reduction in chicken colonization observed in protection experiments varies between 6 log_10_ to 1 log_10_ when compared to control groups (Wyszyńska et al., 2004; Buckley et al., 2010; Layton et al., 2011; Theoret et al., 2012). However, those various experiments differ substantially. Comparison of the experiments is difficult, if not impossible, because of differences in the nature of the antigens, the routes of antigen administration, the use of adjuvant, and the schemes of immunization or challenge experiments. Additionally, recent progress in chicken genome sequencing has revealed enormous differences among commercial breeds of broiler chickens (Rubin et al., 2010; Yan et al., 2014; Yi et al., 2014). These differences may have an impact on the bird immune system functioning and on colonization by *Campylobacter* (Humphrey et al., 2014). Differences in the immune responses to infection were observed not only among various breeds of chickens but also between among individual birds of the same population (Connell et al., 2012), which may explain the varying levels of colonization observed between individual birds in our protection experiments (see **Figure 8**). Also, the gastrointestinal microbiome of chickens differs for genetically diverse birds (Oakley et al., 2014; Schokker et al., 2015). Thus, in the light of the knowledge from the global analysis of chicken genomes or transcriptomes, it is apparent that the chicken line is of great importance when vaccine prototypes are evaluated. Also, the presence of maternal antibodies should be taken into account when results of immunization are evaluated. To exclude the impact of maternal antibodies, some experiments have been conducted on SPF chickens (Buckley et al., 2010; Hodgins et al., 2015).

Many recent experiments have sought to clarify how the location of antigen delivered by LAB strains affects the efficacy of vaccination (Reveneau et al., 2002; Wyszyńska et al., 2015). Generally, surface located antigens work more efficiently than those located in the cytoplasm; however, some exceptions were also observed (Shaw et al., 2000). Thus, this issue needs to be evaluated individually for each antigen and delivery vector. Our protection experiments demonstrated that the hybrid protein, equipped with a cell-wall anchored motif and delivered orally using *L. lactis* as a vehicle, acted more effectively than cytoplasm-located protein administered by the same strain. Also, using the hybrid rCjAD protein resulted in a higher level of protection when compared to surface-located CjaA. It should be noted that one of the CjaD epitopes inserted into CjaA was the same as that used by Layton et al. (2011). However, Layton et al. (2011) demonstrated a much higher level of protection. The reasons for the discrepancy are difficult to identify as both vaccination experiments differ substantially. One notable difference is that the *Salmonella* strain used by the Layton group as the carrier strain co-expressed the immune-enhancing region of the CD154 ligand (Layton et al., 2011).

Overall, our work demonstrates the possibility of delivering foreign antigens via an LAB vector for chicken immunization against *Campylobacter*, and it also documents that CjaA is a good starting point for constructing a multi-epitope hybrid protein taking into account recently identified immunogenic *C. jejuni* proteins present in egg yolks of immunized hens (Hermans et al., 2014). Hybrid proteins containing epitopes of several immunogenic proteins may ensure higher levels of protection than vaccination with individual proteins.

## Author Contributions

EJ-K, AW, and PK conceived and designed the study. PK, AW, BO, MK, and KD carried out the laboratory work. AW, PK, PM, IA carried out animal experiment. EJ-K, PK, and AW analyzed the data. EJ-K, AW, and PK wrote the manuscript. All authors read and approved the final manuscript.

## Conflict of Interest Statement

The authors declare that the research was conducted in the absence of any commercial or financial relationships that could be construed as a potential conflict of interest.

## Supplementary Material

**FIGURE S1 | The scheme of pUWM1373 construction.**

**FIGURE S2 | Amino acid sequence of CjaA containing inserted three CjaD peptide epitopes (EVSGV, DEWGTDEYN, GETNPVCTEKTKACDAQNRR) in all possible combinations.** Amino acids between which epitopes of CjaD protein were inserted are indicated in red.

**FIGURE S3 | The scheme of pUWM1392 and pUWM1395 construction.**

**FIGURE S4 | The production of CjaA and rCjaAD by *L. lactis*.** Protein extracts were separated by 12% SDS-PAGE under reducing conditions and probed with anti-rCjaAD antibodies. Lanes: M - protein molecular-weight marker, 1 – *L. lactis*/pUWM1392, 2 – *L. lactis*/pUWM1395; 3 – *L. lactis*/pUWM1412.
