# Supplementary figures and images for: Cell Wall Anchoring of the Campylobacter Antigens to Lactococcus lactis
Source: Front Microbiol. 2016 Feb 18;7:165. doi: 10.3389/fmicb.2016.00165 (PMC4757695; doi:10.3389/fmicb.2016.00165)

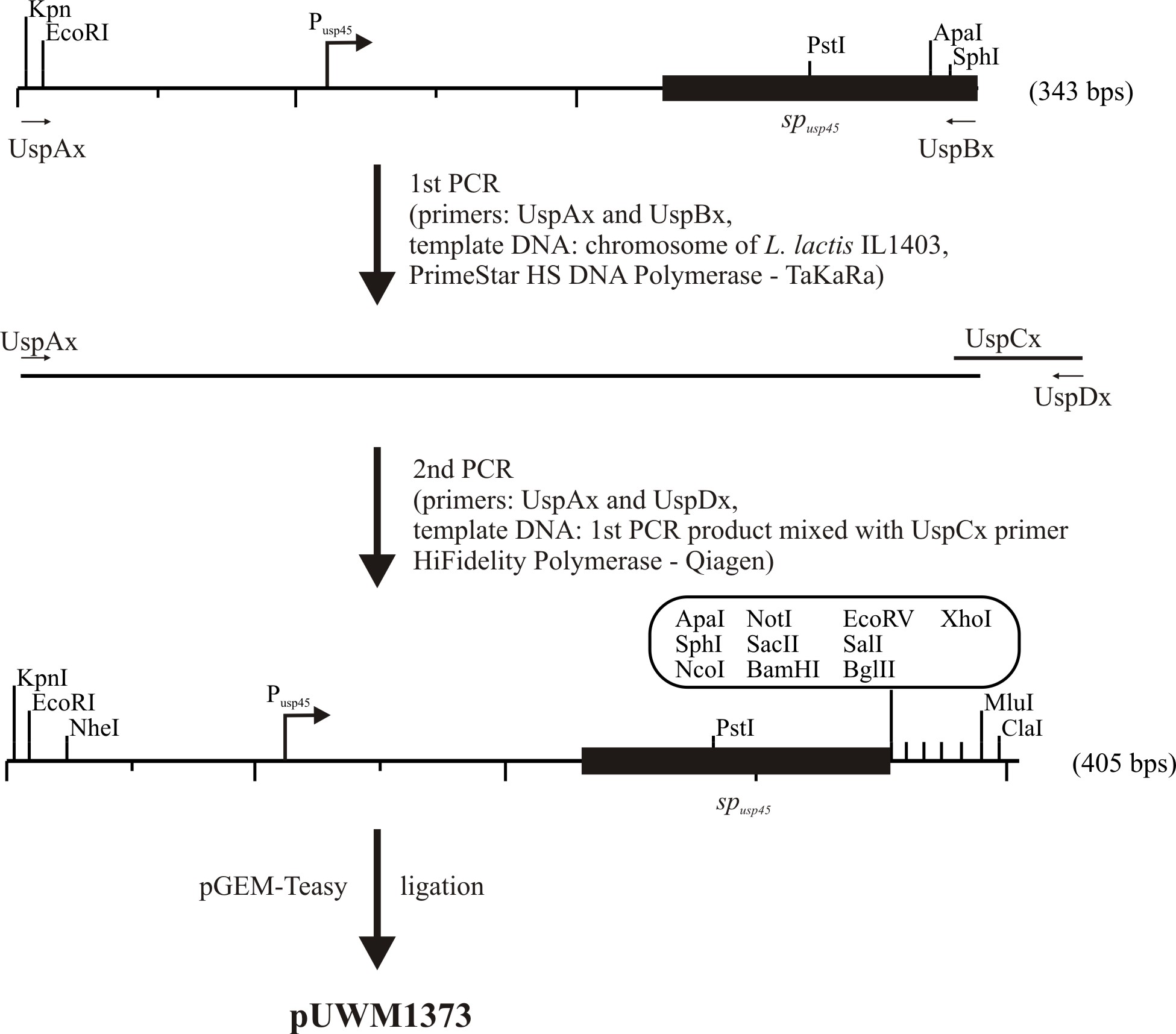

Supplement: Supplementary file 1 [file Image_1.JPEG]

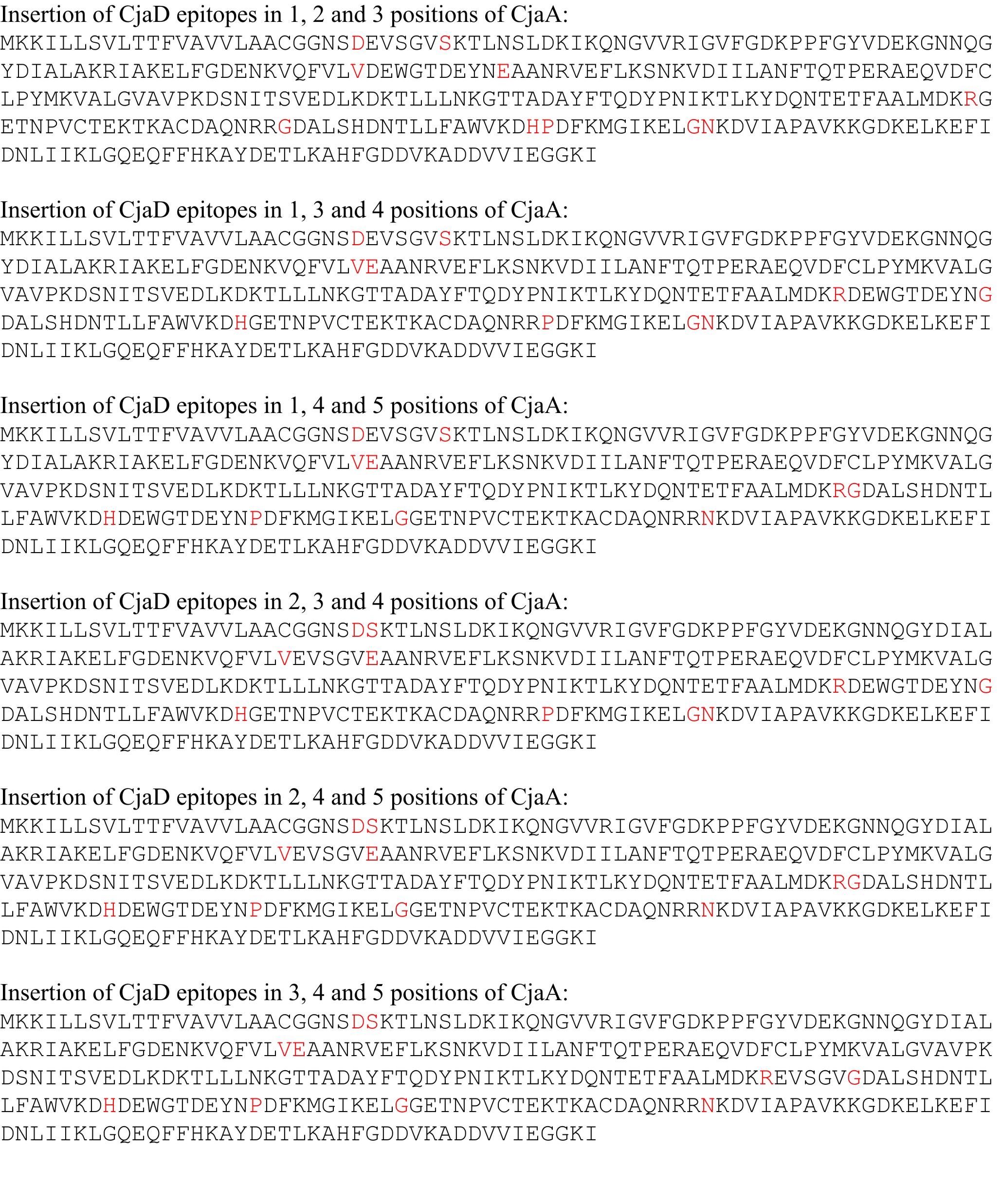

Supplement: Supplementary file 2 [file Image_2.JPEG]

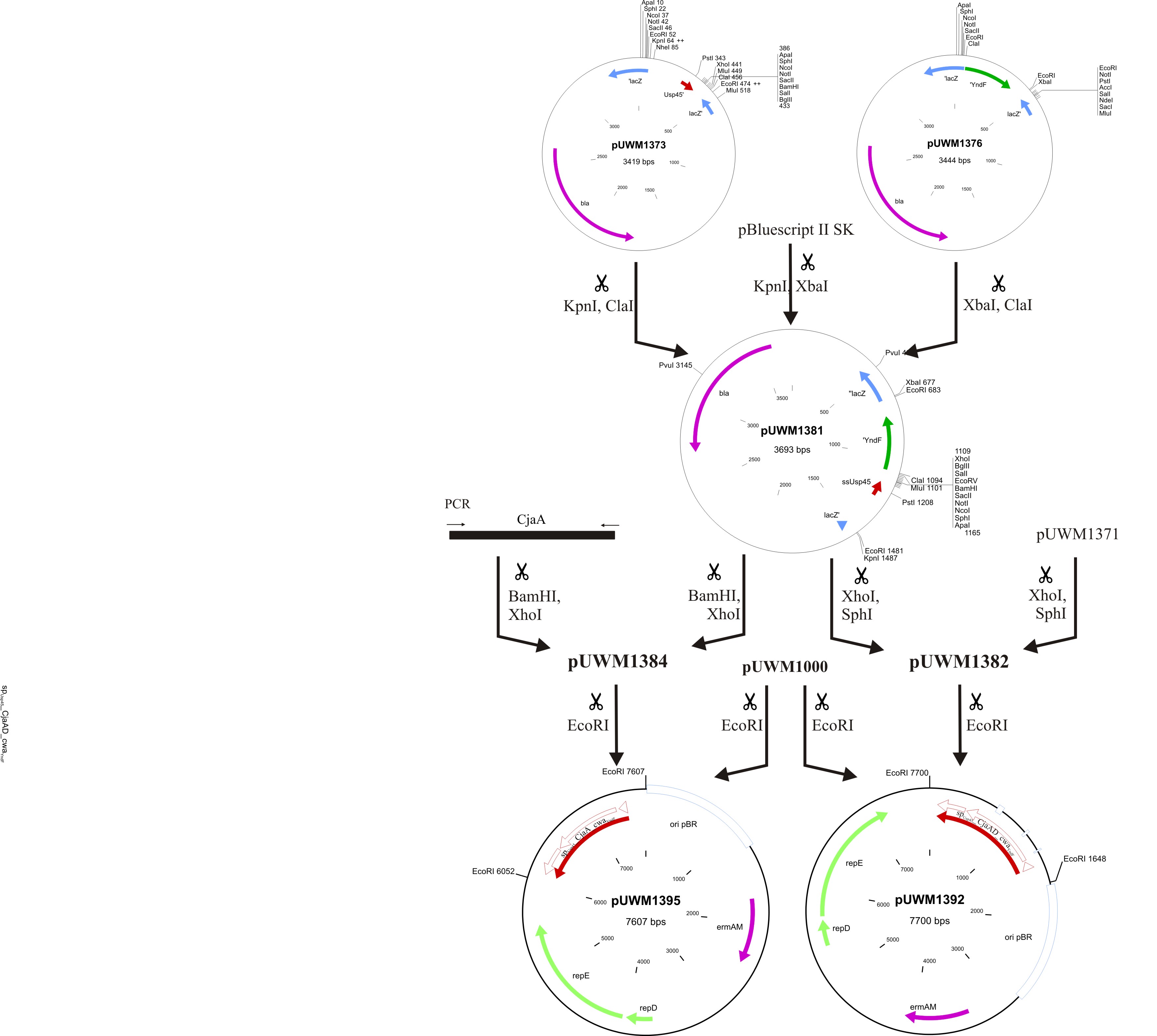

Supplement: Supplementary file 3 [file Image_3.JPEG]

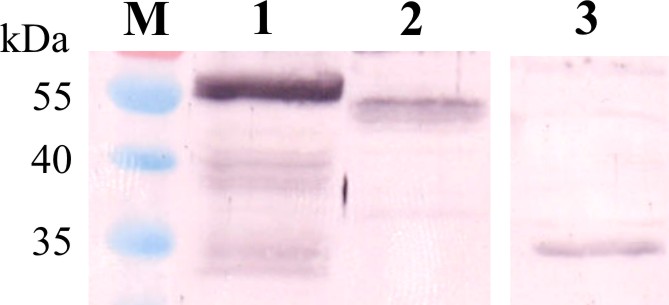

Supplement: Supplementary file 4 [file Image_4.JPEG]
